# Point-of-care ultrasound for the detection of hydronephrosis in emergency department patients with suspected renal colic

**DOI:** 10.1186/s13089-020-00178-3

**Published:** 2020-06-08

**Authors:** Stephanie Sibley, Nathan Roth, Charles Scott, Louise Rang, Heather White, Marco L. A. Sivilotti, Eric Bruder

**Affiliations:** 1 Department of Emergency Medicine, Queen’s University, Kingston, ON Canada; 2 School of Medicine, Faculty of Health Sciences, Queen’s University, Kingston, ON Canada; 3 Department of Radiology, Queen’s University, Kingston, ON Canada

**Keywords:** Hydronephrosis, PoCUS, Point-of-care ultrasound, Renal colic, Emergency department, BMI

## Abstract

**Background:**

Point-of-care ultrasound (PoCUS) by emergency physicians for renal colic has been proposed as an alternative to computed tomography (CT) to avoid ionizing radiation exposure and shorten emergency department length of stay. Previous studies have employed experienced or credentialed ultrasonographers or required advanced ultrasound skills. We sought to measure the diagnostic accuracy of PoCUS by physicians with varied experience using a simplified binary outcome of presence or absence of hydronephrosis. Secondary outcomes include assessment as to whether the presence of hydronephrosis on PoCUS is predictive of complications, and to evaluate possible causes for the reduced diagnostic accuracy such as body mass index (BMI) and time between PoCUS and formal imaging, and scanner experience.

**Results:**

413 patients were enrolled in the study. PoCUS showed a specificity of 71.8% [95% CI 65.0, 77.9] and sensitivity of 77.1% [95% CI 70.9, 82.6]. Hydronephrosis on PoCUS was predictive of complications (relative risk 3.13; [95% CI 1.30, 7.53]). The time interval between PoCUS and formal imaging, BMI, and scanner experience did not influence the accuracy of PoCUS.

**Conclusions:**

PoCUS for hydronephrosis in suspected renal colic has moderate accuracy when performed by providers with varied experience for the binary outcome of presence or absence of hydronephrosis. Hydronephrosis on PoCUS is associated with increased rates of complications. PoCUS for hydronephrosis is limited in its utility as a stand-alone test, however this inexpensive, readily available test may be useful in conjunction with clinical course to determine which patients would benefit from formal imaging or urologic consultation.

*ClinicalTrials.gov Identifier* NCT01323842

## Background

Renal colic is a common presentation in the ED with over 3.6 million visits in the United States between 2006 and 2009 [1]. While most attacks are self-limited, some patients benefit from urologic intervention, and others harbor a more dangerous cause for their pain. Since identifying such cases is largely based on formal imaging, especially computed tomography (CT), suspected renal colic is an important driver of advanced imaging, of prolonged or repeated emergency department utilization, and of direct and indirect costs related to patient care and lost work time [2–4].

Computed tomography remains the imaging modality of choice to confirm the diagnosis, prognosticate spontaneous stone passage, and rule out other causes of abdominal pain [5, 6]. A study of US emergency department visits for suspected renal colic reported very high rates of abdominal CT (> 80%) despite low rates of admission (< 20%) and urologic intervention (< 10%) [7]. Radiology ultrasound (radUS) has been proposed as the preferred test for patients at low risk for complications of urolithiasis [6]. When using this modality, the calculus is often not visualized and the identification of hydronephrosis due to ureteric obstruction becomes the salient finding. Point-of-care ultrasound (PoCUS) by emergency physicians has been widely adopted in emergency medicine [8] and has been proposed as a cost-effective, quick and radiation-free alternative to CT to aid in decision-making in cases of suspected renal colic [9–17]. Several studies have reported only moderate sensitivity and specificity of PoCUS for urolithiasis. These studies have been performed by either credentialed ultrasonographers [12] or attending emergency physicians [15] or have required physicians to perform complicated scans including grading the degree of hydronephrosis and identifying ureteric jets [10, 11, 14, 17]. Smith-Bindman et al. found no significant difference in high-risk diagnoses, return emergency visits, pain scores or hospitalizations when initial ultrasonography was compared to CT. However, there was no head-to-head comparison between PoCUS and CT in this comparative effectiveness study, the diagnosis of renal colic was dependent on passage of a stone or surgical intervention rather than formal imaging, and obese patients were excluded from this study, limiting its generalizability [18].

We performed a prospective, observational study to determine the diagnostic accuracy of PoCUS for hydronephrosis in patients with suspected renal colic. Our secondary outcome evaluated if hydronephrosis on PoCUS was predictive of patients who developed subsequent complications. This study adds to the current body of literature by addressing issues such as physician experience, generalized by having resident and attending emergency physicians with varied levels of ultrasound experience perform scans, and a simplified binary outcome of presence or absence of hydronephrosis as opposed to grading hydronephrosis or detecting ureteric jets. Additionally, the effect of body mass index (BMI), scanner experience and the time interval from formal imaging to PoCUS on the accuracy of this test was assessed.

## Methods

This was a prospective observational study conducted in two academic emergency departments in Ontario (combined visits of ~ 110,000/year). The Queen’s University Health Sciences Research Ethics Board approved this study, which accrued subjects over 30 months (April 2011–July 2013). All subjects provided written consent prior to enrollment.

Patients aged 16–65 years who had either CT or radUS ordered for suspected renal colic were screened for eligibility. The treating emergency physicians enrolled eligible patients 24 h a day, 7 days a week, assisted by dedicated research personnel during daytime to evening hours up to 7 days a week, which matched the availability of advanced imaging for this indication.

Patients were excluded for hemodynamic instability, fever, suspected urinary tract infection based on symptoms or urinalysis positive for leukocytes and nitrites, pregnancy, renal transplant, single functioning kidney, known abdominal aortic aneurysm or incarceration. Patients were ineligible if no formal imaging was ordered at the time of their emergency visit.

Attending or resident emergency physicians performed the PoCUS scans. To be eligible to perform study scans, physicians had to complete an accredited emergency department ultrasound course (such as those endorsed by the Canadian Point of Care Ultrasound Society) or the local, required, introductory point-of-care ultrasound course for emergency medicine residents focusing on focused assessment with sonography in trauma (FAST), aortic and obstetrical ultrasound skills. Scanning physicians had to attend a didactic lecture where the study was described and renal anatomy, ultrasound technique and several examples were reviewed. They attended a training session where physicians scanned live models with hydronephrosis. The newly trained physicians had to complete 25 observed renal ultrasounds with an expert physician (fellowship trained or additional training in renal ultrasound) prior to enrolling patients.

Other than undergoing PoCUS, subjects were treated according to usual practice. The decision and type of formal imaging was left entirely to the discretion of the treating physician, and potential subjects were approached by research staff only after formal imaging had been ordered. Scans were performed using a My Lab 5 (Esaote, Genoa Italy) ultrasound machine with a curvilinear 3.5-MHz probe. Scanning physicians performed a B-mode scan of the abdominal aorta and bilateral kidneys (short and long axis). The scanning physician recorded subject weight, height, if hydronephrosis was present/absent/indeterminate for each kidney, as well as the diameter of the abdominal aorta on a standardized form while blinded to any formal imaging. In cases where the formal imaging was performed prior to PoCUS, the scanning physician was directed not to look at images or the formal radiology report.

A radiologist (CS) blinded to the PoCUS reviewed all formal CT or radUS images to establish the degree of hydronephrosis, graded as absent, mild, moderate or severe [19]. The radiologist also documented the presence, position and size of any urinary calculus, signs suggestive of recent stone passage, as well as any alternative diagnosis that could account for the patient’s symptoms and the presence of abdominal aortic aneurysm (AAA) on a standardized form.

Research assistants performed 30-day telephone follow-up and asked scripted standardized questions regarding urgent consultations with urologists, any interventions or hospitalizations. A chart review was performed at 30 days (NR and SS). Details of urologic consultations, interventions, hospitalizations, sepsis and death within the regional healthcare system were extracted from the record, recorded on standardized forms, and entered into a RedCap database. Patients were considered lost to follow-up if they could not be contacted by telephone and had no record of emergency room visit, urology intervention or hospital admission in the medical record.

The primary outcome measure was the sensitivity and specificity of PoCUS for the presence or absence of hydronephrosis compared to formal imaging in patients with suspected renal colic.

The secondary outcome was the association between PoCUS findings and complications within 30 days, defined as the composite of any urologic intervention (e.g., lithotripsy, stent, or percutaneous nephrostomy), sepsis, hospital admission or death. Urgent complications were defined as any of the composite outcomes that occurred within 7 days of initial emergency department visit. Urgent complications were assessed separately to differentiate patients who presented with an acute worsening of their symptoms versus patients who had urology follow-up and were scheduled for a surgical procedure within 30 days. The effects of BMI, time interval to formal imaging and physician scanning experience on the accuracy of PoCUS were analyzed.

For the primary analysis, indeterminate PoCUS scans were deemed negative for hydronephrosis, but reclassified as positive in a separate sensitivity analysis. The degree of severity of hydronephrosis on formal imaging was dichotomized at different cut points to determine the diagnostic accuracy of PoCUS when hydronephrosis was considered positive only when graded “mild, moderate and severe”, “moderate and severe”, or “severe”. Patients who were lost to 30-day follow-up were excluded from the analysis for the secondary outcome of complications and no imputation was performed.

Statistical analysis was performed SAS software, version 9.4 (Cary, North Carolina). Baseline characteristics were summarized as means and standard deviations and medians and quartiles for continuous variables and proportions for binary and categorical variables. Sensitivity, specificity and likelihood ratios were calculated for detection of hydronephrosis with PoCUS using the formal radiology result as the reference standard. A Chi square test was used to compare risk of complications, and to assess for changes in accuracy for detection of hydronephrosis compared to formal imaging time between PoCUS and formal imaging, and BMI.

Results are reported in accordance with the STARD 2015 guidelines for studies of diagnostic accuracy [20].

We performed an a priori sample size calculation using estimates taken from Edmonds et al. [21], a retrospective chart review of patients undergoing formal ultrasound for the diagnosis of renal colic in emergency patients. These investigators reported that 0.6% of subjects with no abnormality on ultrasound received urologic intervention versus 6.2% of patients with a visualized stone and 6.8% of patients with ultrasounds suggestive of ureterolithiasis. Assuming a 7% versus 1% in complications/interventions between patients with and without hydronephrosis on PoCUS, two-tailed α of 5%, and a power of 80%, it was determined that 167 subjects would be required in each group. We estimated that this would provide 95% confidence bands of ± 10% around the point estimate of sensitivity and specificity for the primary outcome.

## Results

**Fig. 1:**
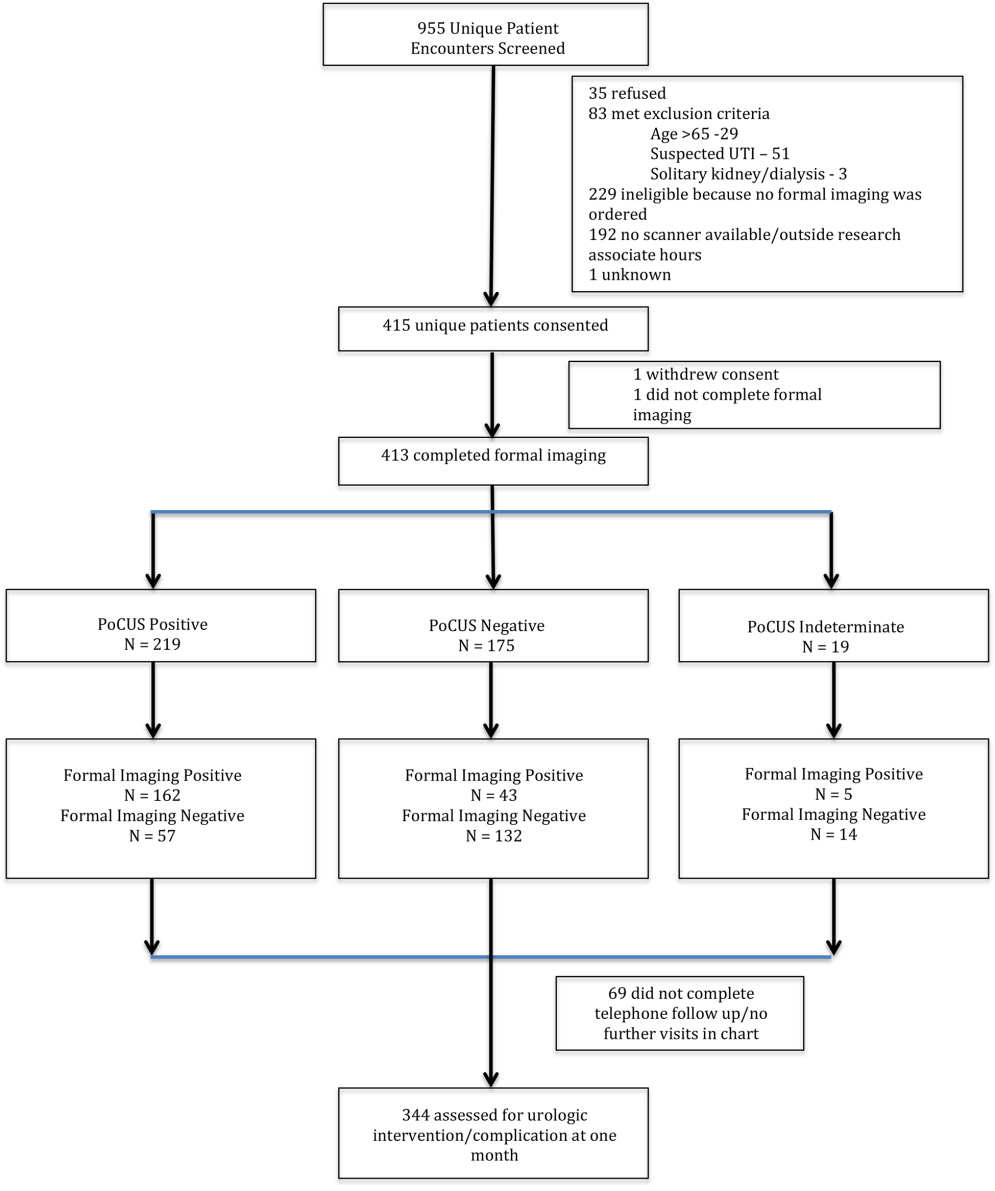
STARD flow diagram of patient enrollment and follow-up

A total of 955 unique patient encounters were screened for potential enrollment in the study. The total enrollment was 415 patients, and 413 patients completed formal imaging and represent the study cohort for the primary analysis of hydronephrosis. Total patients lost at 1-month follow-up totaled 69, leaving 344 patients in the analysis for the secondary outcome of urologic complications (Fig. 1).

Seventeen emergency physicians and residents were eligible to perform renal PoCUS for the study, including five physicians considered to be experts.

Baseline characteristics of the cohort are summarized in Table 1. Half (50.4%) were male, with mean age was 42.2 years and mean BMI was 29 kg/m^2^. Most patients (85.0%) underwent formal imaging with a CT scan. Hydronephrosis on formal imaging was found in 51.0% of patients. Hydronephrosis on PoCUS was seen in 53.0% of patients, and 4.6% had indeterminate PoCUS scans. A small number of subjects had alternate diagnoses established on formal imaging, including ovarian cysts, diverticulitis and appendicitis (Table 2). Of those with an alternate diagnosis the detection of hydronephrosis with POCUS was rare and seen only in one case of pyelonephritis, one case of perforated diverticulitis, one ureteric stricture and one gynecological cancer. There were no cases of ectopic pregnancy or abdominal aortic aneurysm in the study cohort.

**Table 1:** Cohort characteristics

N	413
Male (%)	208 (50.4)
Age, years mean (SD)	42.2 (13.5)
BMI, mean (SD)	28.96 (11.2)
BMI category (%)	
< 20	27 (6.7)
20–24.9	111 (27.6)
25–29.9	126 (31.3)
30–34.9	76 (18.9)
> 35	62 (15.4)
Missing	10
Time from POCUS to formal imaging, min	
Mean (SD)	−31.81 (63.1)
Median	−40.50
25th percentile	−58.0
75th percentile	−24.0
Hydronephrosis present on POCUS	
No	175 (42.4)
Yes	219 (53.0)
Indeterminate	19 (4.6)
Formal imaging modality	
CT	350 (85.0)
US	63 (15.0)
Hydronephrosis on formal imaging	
Severe	19 (4.6)
Moderate	78 (19.0)
Mild	113 (27.4)
Absent	202 (49.0)
2nd ER visit for Imaging results (%)	49 (12)
Urologic Consult	60 (17.4)
Urologic intervention/complication	
Lithotripsy	15 (4.4)
Stent	20 (5.8)
Percutaneous nephrostomy	0
Sepsis	8 (2.3)
Hospital admission	17 (5.2)
Death	0
Stone or evidence of recent passage seen on formal imaging (%)	205 (50)

*SD* standard deviation, *BMI* body mass index, *CT* computed tomography, *US* ultrasound)

**Table 2:** Alternative diagnoses found on formal imaging

Ovarian cyst	6
Diverticulitis	4
Appendicitis	3
Pyelonephritis	3
Biliary colic/cholecystitis	3
Ovarian torsion/mass	2
Bony lesions/compression fracture	2
Epiploic appendagitis	2
Shingles	2
Uterine mass/cancer	2
Testicular torsion	1
Interabdominal carcinomatosis with metastatic disease to the liver	1
Pneumonia	1
Bowel obstruction	1
Ileitis	1
AAA	0
Ectopic pregnancy	0

*AAA* abdominal aortic aneurysm

Twenty-eight (6.8%) subjects developed the composite outcome at 30 days, with 19 (4.6%) deemed to be urgent within 7 days. There were no deaths.

The sensitivity of POCUS for the detection of hydronephrosis was 77.1% [95% CI 70.9, 82.6] and the specificity was 71.8% [95% CI 65.0, 77.9]. The sensitivity of PoCUS improved with worsening degrees of hydronephrosis. Two unplanned sensitivity analyses were performed. These measures did not improve appreciably for patients with confirmed stone or signs of recent stone passage on formal imaging (sensitivity 78.2 [95% CI 71.3, 84.1], specificity 74.2 [95% CI 55.4, 88.1]), or when considering only patients who had a CT scan (sensitivity 75.9 [95% CI 69.2, 81.8], specificity 72.3 [95% CI 64.7, 79.1]) (Table 3).

**Table 3:** Sensitivity, specificity, positive-likelihood ratio and negative-likelihood ratio for detection of hydronephrosis with POCUS

	TP	TN	FP	FN	Sensitivity (95% CI)	Specificity (95% CI)	+LR (95% CI)	−LR (95% CI)
Hydronephrosis (mild/moderate/severe positive)^a^	162	146	57	48	77.1% (70.9, 82.6)	71.8% (65.0, 77.9)	2.73 (2.17, 3.48)	0.31 (0.24, 0.41)
Hydronephrosis (moderate/severe positive)^a^	85	182	134	12	87.6 (79.4, 93.4)	57.5 (51.8, 63.0)	2.05 (1.78, 2.39)	0.22 (0.13, 0.37)
Hydronephrosis (severe positive)^a^	18	193	201	1	94.7 (74.0, 99.9)	48.9 (43.8, 53.9)	1.85 (1.60, 2.14)	0.11 (0.02, 0.73)
Hydronephrosis (with confirmed stone on formal imaging)^b^	136	23	8	38	78.2 (71.3, 84.1)	74.2 (55.4, 88.1)	3.03 (1.66, 5.53)	0.29 (0.21, 0.42)
Hydronephrosis (patients undergoing CT scan)^c^	145	115	44	46	75.9 (69.2, 81.8)	72.3 (64.7, 79.1)	2.74 (2.11, 3.57)	0.33 (0.25, 0.44)

*LR* likelihood ratio, *TP* true positive, *TN* true negative, *FP* false positive, *FN* false negative)

^a^A positive test on formal imaging was dichotomized at various cut points (mild/moderate/severe, moderate/severe, severe)

^b^Sensitivity analysis with confirmed stone on formal imaging

^c^Senstivity analysis with the gold standard of CT scan

The presence of hydronephrosis on PoCUS was associated with increased complications (11.8% [95% CI 7.6, 17.4] versus 3.8% [95% CI 1.4, 8.0], relative risk of 3.1 [95% CI 1.3, 7.5]) (Table 4). The association between hydronephrosis on formal imaging and 30-day complications was somewhat stronger (14.5% [95% CI 9.7, 20.6] versus 1.21% [95% CI 0.2, 4.3], relative risk 11.98 [95% CI 2.9, 49.7]).

**Table 4:** Intervention/complication for POCUS and formal imaging

	Intervention/complication	No intervention/ complication	Risk (95% CI)	Relative risk (95% CI)
POCUS				
Positive	22	163	11.83% (7.56, 17.36)	3.13 (1.30, 7.53)
Negative/indeterminate	6	153	3.77% (1.40, 8.03)	
POCUS				
Positive/indeterminate	23	179	11.39% (7.36, 16.59)	3.23 (1.26, 8.30)
Negative	5	137	3.52% (1.15, 8.03)	
Formal imaging				
Positive	26	153	14.53% (9.71, 20.55)	11.98 (2.89, 49.70)
Negative	2	163	1.21% (0.15, 4.31)	

Patients were positive for intervention/complication if they had any one of the following at 30 days follow-up—lithotripsy, stent, percutaneous nephrostomy, sepsis, hospital admission or death

In a post hoc analysis, we considered only the subgroup of patients with urgent interventions. The presence of hydronephrosis on PoCUS was also associated with urgent interventions within 7 days (8.1% [95% CI 4.6, 13.0] versus 2.5% [95% CI 0.7, 6.3], relative risk of 3.2 [95% CI 1.1, 9.5]).

A sensitivity analysis was performed where indeterminate scans were considered positive, with little difference in complications (11.4% [95% CI 7.0, 15.7] vs 3.5% [95% CI 0.4, 6.6], relative risk 3.23% [95% CI 1.3, 8.3]).

**Fig. 2:**
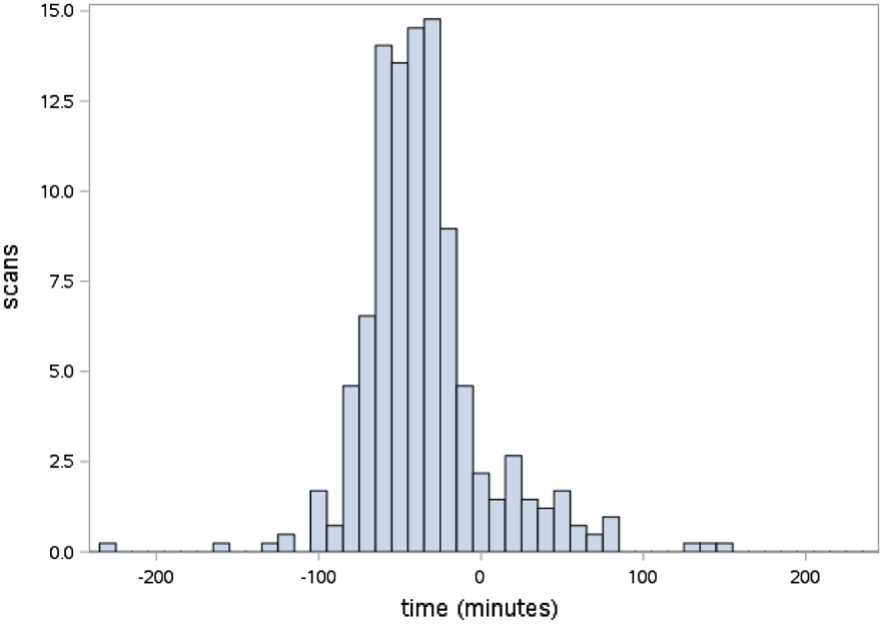
Time distribution of POCUS to formal imaging (time = 0 − time of PoCUS exam)

The diagnostic accuracy of PoCUS for hydronephrosis did not appear to be strongly influenced by any of the pre-specified factors we examined. Almost all PoCUS scans were obtained within 1 h of formal imaging (Fig. 2). Most (86.2%) patients had PoCUS performed after being transferred back to the emergency department from formal imaging. The agreement did not improve when the absolute value of the time interval between formal imaging and PoCUS was shorter. Agreement with formal imaging for each quartile was 76.2%, 78.1%, 76.5% and 67.3% (*p* = 0.27).

**Fig. 3:**
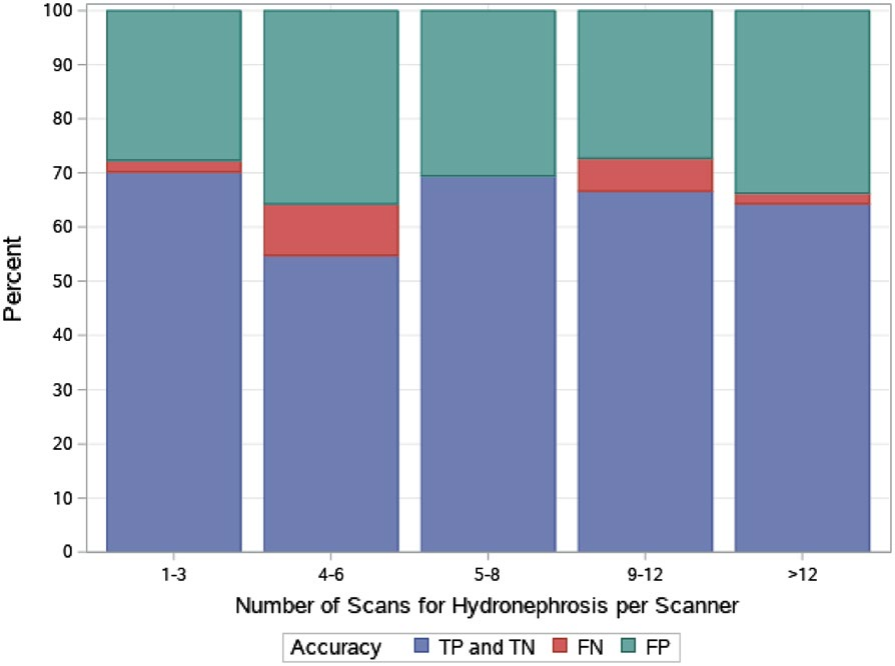
Accuracy of POCUS with formal imaging with increasing scanning experience (*TP* true positive, *TN* true negative, *FP* false positive, *FN* false negative)

When performing PoCUS for the detection of hydronephrosis, expert scanners were comparable to non-experts (sensitivity 81.5% [95% CI 70.0, 90.1] vs 75.2% [95% CI 67.3, 82.0]), (specificity 65.4 [95% CI 50.9, 78.0] vs 74.2 [95% CI 66.4, 80.9]). There was no difference in agreement with formal imaging, with experts in agreement 66.7% [95% CI 57.4, 75.1] and non-experts in agreement 63.9% [95% CI 58.1, 69.3] (*p* = 0.65). Agreement between PoCUS and formal imaging did not improve with a physician’s increased scanning experience within the study Agreement was 70.1% for 1–3 scans, 54.8% for 4–6 scans, 69.4% for 10–12 scans, and 64.3% for > 12 scans (Fig. 3).

**Fig. 4:**
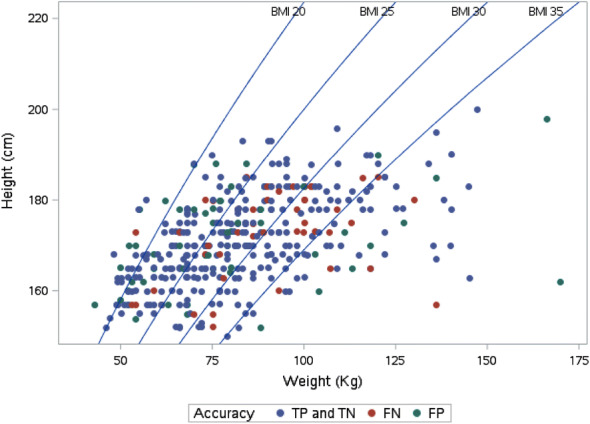
Accuracy of POCUS for the detection of hydronephrosis relative to patient BMI (*TP* true positive, *TN* true negative, *FP* false positive, *FN* false negative)

BMI did not influence the accuracy with agreement between PoCUS and formal imaging of 70.4%, 73.9%, 72.2% and 81.6% and 71.0% for BMI of < 20, 20–24.9, 25–29.9, 30–34.9 and > 30, respectively (*p* = 0.56) (Fig. 4).

## Discussion

Our study reaffirms the modest sensitivity and specificity reported by previous studies which limits the utility of PoCUS as a stand-alone diagnostic tool for renal colic. PoCUS is predictive of complications and may help identify patients who require further imaging and urologic consultation.

Our study has similar results to a meta-analysis [9] by Wong et al. that demonstrated modest diagnostic accuracy for nephrolithiasis with moderate sensitivity (77.1%) and specificity (71.8%). They included high-quality studies (*N* = 1773) including two studies with scans performed by credentialed ultrasonographers [12] or attending emergency physicians [15] and four studies requiring physicians to perform more complicated scans [10, 11, 14, 17]. While these studies showed improved specificity, physician ability to grade hydronephrosis is moderate and limits sensitivity [22]. Our study demonstrated that physicians at all levels of training and experience can obtain similar sensitivity and specificity while performing a simplified scan for the presence or absence of hydronephrosis.

The presence of hydronephrosis on PoCUS was associated with an increased risk of subsequent urologic intervention or complication in our study. Fields et al. [11] performed a study of 77 emergency department patients to predict 30-day outcomes. Thirteen patients required admission to hospital, and all admitted patients had hydronephrosis on their PoCUS exam. Daniels et al. [10] performed a study to determine the ability of PoCUS to predict need for urologic intervention at 90 days and demonstrated that 22.8% of patients with hydronephrosis as opposed to 10% of those without required urologic intervention.

While PoCUS was associated with need for urologic intervention, it identified fewer patients than CT or radUS. This is in keeping with a study by Ganesan et al. suggesting 22% of patients would be inappropriately counseled for either observation or intervention if ultrasound was used alone to guide clinical decision-making [23].

Smith-Bindman et al. showed PoCUS has the advantage of being a readily available modality that has the potential to reduce the time from presentation to imaging in the emergency department and expedite clinical decision-making [18]. In this study, we hypothesized the modest sensitivity and specificity of PoCUS in previous studies may have been due to the length of time between PoCUS and formal imaging due to factors such as dehydration and fluid administration [24], and spontaneous stone passage [25]. PoCUS was performed as close to the formal imaging as possible to reduce the effect of these factors. Most of the PoCUS scans were performed within 1 h of formal imaging and there was no difference in agreement with shorter time intervals, indicating these factors had little influence on the sensitivity and specificity in this study.

There was no difference in accuracy between expert and non-expert scanners and no improvement with increased scanning experience which suggests the training session and 25 practice scans prior to enrolling patients in the study may be adequate to develop and retain the skill for detection of hydronephrosis. This is in contrast to Herbst et al. who found fellowship trained emergency physicians had greater accuracy than those without fellowship training [14]. This difference is likely a result of the differing definition of expert scanners and the outcome of graded hydronephrosis versus a simplified binary outcome. It is important to note the overall sensitivity (72.6%), specificity (73.3%) were similar.

Few PoCUS studies have assessed the relationship between diagnostic accuracy and BMI for detection of renal colic. BMI was not a predictor of accuracy of PoCUS for hydronephrosis in this study, and good-quality images were obtained in patients with BMI up to 35 kg/m^2^. This is in keeping with findings using ultrasound by diagnostic imaging sonographers [23, 26], who found that detection of a renal stone was independent of BMI.

While PoCUS for hydronephrosis lacks the sensitivity and specificity for a stand-alone diagnostic test, it may be useful when combined with a clinical prediction tool to improve accuracy in diagnosis [10] or clinical score based on history and physical exam [27]. The ability to use this test in patients with increased risk factors such as older age, fever, leukocytosis, history of kidney disease or solitary kidney remains unclear.

### Strengths and limitations

The strengths of this study are the inclusion of both resident and attending physicians with varied PoCUS experience and our comprehensive renal PoCUS training program which included practice scanning on patients with known hydronephrosis and observed scans by PoCUS experts. This study also included obese patients who were excluded from other large studies.

Limitations to our study include missed, potentially eligible patients due to lack of an available scanning physician which may have introduced a selection bias. As well, many patients had their PoCUS exam performed after their formal imaging was completed, however scanners were instructed not to look at any imaging prior to completing the PoCUS exam. Most of the PoCUS exams were completed within 1 h of the formal imaging, and it is unlikely the formal reports would be available prior to the completion of the PoCUS exam, mitigating this risk of unblinding. Finally, radUS is not considered a diagnostic gold standard for detection of renal stones or recent passage. 15% of patients had formal ultrasound as opposed to CT scanning, mainly young women. A sensitivity analysis was performed for patients who received a CT scan and little difference in sensitivity and specificity was noted.

## Conclusion

PoCUS for hydronephrosis has moderate sensitivity and specificity for renal colic limiting its utility as a diagnostic test. However, patients with hydronephrosis are more likely to develop complications, and PoCUS may be useful to help guide further imaging and consultation in conjunction with clinical course. BMI, provider experience and time between PoCUS and formal imaging do not influence the accuracy of this test.

## Data Availability

All data collection forms are stored in the Department of Emergency Medicine Research Office. The RedCap database used for electronic entry is housed at Queen’s University Clinical Evaluation Research Unit. This data is not publicly available due to confidentially issues, however, de-identified data are available from the corresponding author on reasonable request.
